# XBP1 gene silencing augments quercetin-induced apoptosis and anti-metastatic activity in breast cancer cell lines

**DOI:** 10.1007/s13205-026-04923-8

**Published:** 2026-06-17

**Authors:** Sedef Akçaalan, Canan Eroğlu Güneş, Ercan Kurar

**Affiliations:** 1 Department of Medical Biology, Faculty of Medicine, Necmettin Erbakan University, Konya, Türkiye Turkey; 2 Department of Molecular Biology and Genetics, Faculty of Science, Necmettin Erbakan University, Konya, Türkiye Sedef Akçaalan Turkey

**Keywords:** Apoptosis, Breast cancer, EMT, Metastasis, shRNA, XBP1

## Abstract

This study investigated the combined effects of quercetin (Que) and shRNA-mediated XBP1 silencing on the MCF7 and MDA-MB-231 breast cancer cell lines. Que was selected because of its well-known anticancer effects. XBP1, on the other hand, was targeted because it has been reported to play a critical role in the progression of breast cancer and in stress-adaptive signaling. Cell viability and colony formation assays showed that Que and XBP1 silencing reduced cell growth, with the combined treatment producing a more pronounced inhibitory effect. In both cell lines, the combined treatment was associated with increased expression of apoptosis-related markers, including CASP3, CASP8 and CASP9, supporting a pro-apoptotic response. In addition, treatment-related changes were observed in EMT-associated markers, including increased CDH1 expression and decreased CDH2 expression, suggesting modulation of EMT-related molecular characteristics. Overall, these findings indicate that the combined application of XBP1 silencing and Que exerts anti-proliferative and apoptosis-associated effects on breast cancer cells and may also influence EMT-related molecular pathways. These results support the potential of combining RNAi-based approaches with plant-derived natural compounds as a therapeutic strategy for breast cancer.

## Introduction

Breast cancer is the most commonly diagnosed malignancy among women and remains one of the leading causes of cancer-related mortality worldwide (Siegel et al. 2023). In the United States alone, 2,041,910 new cancer cases and 618,120 cancer-related deaths are estimated for 2025 (Siegel et al. 2025). Based on molecular and histopathological features, breast cancer is classified into three major subtypes: estrogen and/or progesterone receptor-positive (ER+/PR+), HER2-overexpressing, and triple-negative breast cancer (TNBC), which lacks expression of ER, PR, and HER2 (Barzaman et al. 2020; Zhang 2023). These subtypes exhibit significant differences in biological behavior, prognosis and response to treatment. Among them, TNBC represents a major therapeutic challenge due to its aggressive phenotype, high metastatic potential and limited targeted therapy options (Bianchini et al. 2016). Consequently, the development of alternative therapeutic strategies that modulate apoptosis and epithelial-mesenchymal transition (EMT) pathways through the use of natural compounds and gene-targeting approaches holds promise for improving breast cancer treatment.

Flavonoids are phenolic compounds found in various parts of plants, recognized for their antioxidant, anti-inflammatory and anticancer properties (Erlund 2004; Harborne and Williams 2000; Middleton et al. 2000). Quercetin (Que), a polyphenol belonging to the flavonol subclass, is abundantly present in foods such as onions, apples and tea (Deepika and Maurya 2022; Singh et al. 2021). Its antioxidant activity primarily stems from free radical scavenging and metal ion chelation (Hanasaki et al. 1994; Murota and Terao 2003). Moreover, Que has exhibited anticancer effects in various tumor models by arresting the cell cycle, inducing apoptosis and inhibiting angiogenesis (Sak 2014; Yang et al. 2020). However, the effects of Que on endoplasmic reticulum (ER) stress remain limited. Elevated ER stress associated with HSP90 inhibition leads to activation of mitochondria-mediated apoptotic pathways, resulting in the activation of key caspases such as CASP3 and CASP9 (Aalinkeel et al. 2008; Vargas and Burd 2010). The modulatory role of Que in these pathways has generated interest in its potential as a targeted therapeutic agent in cancer treatment.

X-box binding protein 1 (XBP1) was first cloned in 1990 and identified as a unique bZIP transcription factor capable of binding to the X-box element located in the promoters of human MHC class II genes (Liou et al. 1990). XBP1 functions downstream of inositol-requiring enzyme 1 alpha (IRE1α) and plays a key role in the unfolded protein response (UPR), a cellular mechanism that promotes survival under stress conditions. The UPR is initiated in the ER through three membrane-bound signaling pathways including IRE1α-XBP1, PERK-ATF4 and ATF6. It restores homeostasis disrupted by the accumulation of misfolded proteins caused by stressors such as hypoxia or glucose deprivation (Chen et al. 2020; Walter and Ron 2011). Upon activation, IRE1α mediates splicing of XBP1 mRNA, producing the spliced isoform of XBP1, which acts as a potent transcriptional activator (Chen et al. 2020; Hu et al. 2015). XBP1 regulates the expression of ER chaperones and proteins involved in ER-associated degradation (ERAD), thus playing a central role in maintaining ER homeostasis and promoting cell survival. However, when ER stress is prolonged and exceeds the capacity of the UPR, pro-apoptotic signaling pathways are activated, leading to cell death (Ma et al. 2014). Aberrant expression of XBP1 has been linked to apoptosis, inflammation, and increased susceptibility to oxidative stress in various pathological conditions (Li et al. 2009; Ma et al. 2014; Ryoo et al. 2007; Zhong et al. 2012). Additionally, XBP1 is involved in regulating metabolic processes including lipid and glucose metabolism (Wang et al. 2017). During cancer development and progression, tumor cells experience ER stress due to oncogene activation and metabolic challenges, which may activate the UPR and support tumor viability and growth (Harnoss et al. 2020). XBP1 plays a critical role in tumor growth, metastasis, and epithelial-mesenchymal transition (EMT) through interactions with multiple signaling pathways across various cancer types (Chen et al. 2020). In breast cancer, overexpression of XBP1 has been associated with endocrine resistance (Clarke and Cook 2015). Under hypoxic conditions, XBP1 interacts with HIF1α, promoting tumor angiogenesis and progression (Chen et al. 2014). Silencing of XBP1 has been shown to enhance bortezomib sensitivity and inhibit tumor growth in multiple myeloma (Leung-Hagesteijn et al. 2013; Wang et al. 2017). Moreover, XBP1 promotes cancer cell invasion and metastasis by regulating the expression of EMT-related transcription factors (Cuevas et al. 2017; Li et al. 2015; Wu et al. 2018). These findings highlight the role of XBP1 as a critical regulator of tumor survival, proliferation, and metastatic potential (Chen et al. 2020).

Apoptosis is a fundamental process essential for morphogenetic homeostasis throughout life, from early development to adulthood. It ensures the removal of unwanted or damaged cells in a genetically controlled manner and plays a critical role in immune responses and tissue remodeling following injury. Various internal and external stimuli, such as oxidative stress, DNA damage, or hypoxia, can trigger apoptosis in different cell types. Notably, cancer therapies like radiation and chemotherapy often induce apoptosis through p53-dependent DNA damage pathways (Kashyap et al. 2021). However, in pathological conditions such as cancer, cells may evade apoptosis, leading to uncontrolled proliferation. Cancer cells frequently overexpress anti-apoptotic proteins, tipping the balance between pro- and anti-apoptotic members of the Bcl-2 family in favor of survival (Korsmeyer et al. 1993). Consequently, many anticancer treatments aim to induce apoptosis in malignant cells by reactivating apoptotic signaling pathways (Mohammad et al. 2015).

Metastasis is a complex, multi-step process in which tumor cells detach from the primary tumor and disseminate to distant tissues and organs. This process involves various mechanisms including cell invasion, evasion of immune surveillance and remodeling of the tissue microenvironment. Metastasis is a leading cause of treatment failure in many cancer types (Bakir et al. 2020). EMT plays a critical role in metastasis by enabling cells to cross the basal membrane and acquire a mesenchymal phenotype that supports migration (Kalluri and Weinberg 2009). EMT is a dynamic and reversible process essential for embryogenesis, wound healing and pathological conditions such as fibrosis and cancer metastasis (Bakir et al. 2020; Park et al. 2022). During EMT, epithelial cells lose their polarity and cell–cell adhesion properties while acquiring mesenchymal characteristics. This transition is marked by the decreased expression of epithelial markers such as E-cadherin and increased expression of mesenchymal markers like vimentin and N-cadherin, which collectively enhance cell motility, invasiveness, extracellular matrix (ECM) degradation, and tumor dissemination (Chao et al. 2010; Huang et al. 2022; Kalluri and Weinberg 2009).

Given these findings, this study aims to investigate the effects of Que treatment, shRNA-mediated XBP1 gene silencing, and their combination on apoptosis, EMT and metastasis-related biological processes on breast cancer cells.

## Materials and methods

### Cells and culture conditions

Human breast cancer cell lines MCF7 (ATCC^®^ HTB-22™) and MDA-MB-231 (ATCC^®^ HTB-26™) were purchased from the American Type Culture Collection (ATCC, USA). The cell lines were cultured in RPMI 1640 medium (Biowest) supplemented with 10% heat-inactivated fetal bovine serum (FBS, Gibco) and 1% penicillin-streptomycin (Gibco), under standard conditions of 37 °C in a humidified incubator with 5% CO_2_. The cells were subcultured every three days to maintain optimal growth conditions.

### Transduction of MCF7 and MDA-MB-231 cells

Human XBP1 gene-specific miRNA-adapted shRNA sequences were obtained as distinct viral stocks of GIPZ lentiviral particles. These stocks correspond to the V2LHS_18216 (Antisense: TTCACTACCACATTAGCTT) and V3LHS_387388 (Antisense: TAGACACTAATCAGCTGGG) clones (Dharmacon, UK). The relative transduction efficiency of the cells used in the study was determined using GIPZ non-silencing control viral particles. Subsequently, MCF7 and MDA-MB-231 cells were transduced according to the manufacturer’s recommendations. Cells were seeded into 24-well plates at a density of 5 × 10⁴ cells/well and incubated at 37 °C in a 5% CO_2_ incubator for 24 h. At the end of this period, the medium was removed and replaced with 225 µL of serum-free medium containing 25 µL of pre-diluted viral particles (1:25). The cells were incubated for 4 h at 37 °C in a CO_2_ incubator, followed by the addition of 1 mL complete medium. The cells were cultured for an additional 48 h and then analyzed for the expression of the reporter gene TurboGFP using fluorescent microscopy. Puromycin selection was performed to eliminate non-transduced cells. Puromycin-containing medium (2 µg/mL for MCF7 and MDA-MB-231) was added, and the selective medium was replaced every 2–3 days for 10–14 days for selection of transduced cells.

### Fluorescence-based imaging of transduced cells

The expression of the TurboGFP reporter gene in transduced cells was evaluated using fluorescence imaging with a Zeiss Colibri 7 fluorescent microscope (Germany). Excitation was set to 555 nm, and the emission was measured at 475 nm. Imaging was performed to capture fluorescence signals, after which cells and colonies were counted manually under fluorescence microscopy to quantify transduction efficiency.

### Que treatment and cell viability assay

The XTT [2,3-bis(2-methoxy-4-nitro-5-sulfophenyl)-2 H-tetrazolium-5-carboxanilide] assay was performed to evaluate the cytotoxic effects of Que, shRNA transductions, and combined treatments. Briefly, MCF7 and MDA-MB-231 cells were seeded at a density of 10³ cells per well in 96-well plates. To assess the cytotoxic activity of Que, cells were treated with increasing concentrations of Que (0-200 µM) diluted in RPMI-1640 medium. To evaluate the effects of cell transduction and combined therapy on cell viability, cells were treated with Que at the previously determined IC_50_ concentration. The XTT assay was performed following 24, 48, and 72 h of incubation. After the addition of 50 µL XTT solution to each well, the plates were incubated for an additional 4 h in the dark at 37 °C. Optical density was measured at 450 nm (reference wavelength: 630 nm) using a microplate reader. Cell viability was calculated using the formula, Viability (%) = (Absorbance of experimental well / Absorbance of control well) × 100. Each treatment condition was analyzed in triplicate wells, and the experiment was independently repeated three times.

### Combined treatment

In the previous section, MCF7 and MDA-MB-231 cells transduced with silenced XBP1 were seeded in 6-well plates and incubated in a CO_2_ incubator at 37 °C for 24 h. Cells were allowed to acclimate for 24 h after seeding prior to treatment in order to ensure uniform attachment, recovery from seeding stress, and stabilization of basal growth conditions. After incubation, the medium was removed, and 3 mL of fresh medium was added to the control group. However, 3 mL of medium containing Que at the dose determined by cytotoxicity analysis was added to the treatment group. For the combined treatment group, the cells were incubated under the same conditions for an additional 48 h. The effectiveness of Que treatment, XBP1 gene silencing, and their combination was evaluated at the mRNA level by qPCR, at the protein level using western blotting, and in terms of proliferative capacity by colony formation assay.

### Real-time quantitative PCR (qPCR) analysis

MCF7 and MDA-MB-231 cells were cultured in 6-well plates and total RNA was extracted using RiboEx reagent (GeneAll) following the manufacturer’s protocol. RNA samples were treated with DNase-I (Thermo Scientific) to eliminate genomic DNA and reverse-transcribed into cDNA using the iScript cDNA Synthesis Kit (Bio-Rad). qPCR was performed on a Bio-Rad CFX system using SYBR Green dye. The qPCR mix for each gene was prepared in a final volume of 10 µL, containing 5 µL of 2× SYBR Green master mix, 5 pmol of both forward and reverse primers (Table 1) and 2 µL of cDNA. The PCR cycling conditions included an initial denaturation at 95 °C for 10 min, followed by 40 cycles of 95 °C for 30 s, 60 °C for 30 s, and 72 °C for 30 s. Melting curve analysis was used to confirm product specificity. Gene expression levels were normalized to ACTB and CYPA reference genes.

**Table 1:** The primer sequences of target and reference genes used in the qPCR

Gene	Forward primer (5’→3’)	Reverse primer (5’→3’)	Length (bp)	References
BAX	GGAGCTGCAGAGGATGATTG	GGCCTTGAGCACCAGTTT	151	Eroğlu et al. 2018
BCL2	GTGGATGACTGAGTACCTGAAC	GAGACAGCCAGGAGAAATCAA	125	Eroğlu et al. 2018
CASP3	GAGCCATGGTGAAGAAGGAATA	TCAATGCCACAGTCCAGTTC	162	Eroğlu et al. 2018
CASP7	CGAAACGGAACAGACAAAGATG	TTAAGAGGATGCAGGCGAAG	169	Eroğlu et al. 2018
CASP8	GCCCAAACTTCACAGCATTAG	GTGGTCCATGAGTTGGTAGATT	160	Eroğlu et al. 2018
CASP9	CGACCTGACTGCCAAGAAA	CATCCATCTGTGCCGTAGAC	153	Eroğlu et al. 2018
CYCS	GGAGAGGATACACTGATGGAGTA	GTCTGCCCTTTCTTCCTTCTT	102	Eroğlu et al. 2018
FADD	TGACCGAGCTCAAGTTCCTATG	CCAGGTCGTTCTGCTCCAG	108	Eroğlu Güneş et al. 2021
FAS	GTGATGAAGGACATGGCTTAGA	GTGTGCATTCCTTGATGATTCC	156	Eroğlu et al. 2018
TP53	GAGATGTTCCGAGAGCTGAATG	TTTATGGCGGGAGGTAGACT	129	Eroğlu Güneş et al. 2021
CDH1	GAGAGCGGTGGTCAAAGAG	AGCTGGCTCAAGTCAAAGT	117	Eroğlu et al. 2018
CDH2	GCTGACCAGCCTCCAAC	CATGTGCCCTCAAATGAAACC	112	Eroğlu et al. 2018
VIM	TCCAAGCCTGACCTCAC	CACCTGTCTCCGGTACTC	189	Eroğlu Güneş et al. 2021
TIMP1	GCGTTATGAGATCAAGATGACCA	AACTCCTCGCTGCGGTT	141	Eroğlu et al. 2018
TIMP2	GCTGCGAGTGCAAGATCA	CTCTTGATGCAGGCGAAGAA	136	Eroğlu et al. 2018
TIMP3	GCAAGATCAAGTCCTGCTACTAC	GGATGCAGGCGTAGTGTTT	123	Eroğlu Güneş et al. 2021
MMP2	TGGCAGTGCAATACCTGAA	GCATGGTCTCGATGGTATTCT	147	Eroğlu Güneş et al. 2021
MMP9	GCAGACATCGTCATCCAGTT	ACAACTCGTCATCGTCGAAAT	139	Eroğlu et al. 2018
XBP1	CCAGAACATCTTCCCATGGAT	GGGTCCAACTTGTCCAGAAT	89	Eroğlu Güneş et al. 2023
ACTB	AGCACGGCATCGTCACCAACT	TGGCTGGGGTGTTGAAGGTCT	179	Eroğlu Güneş et al. 2021
CYPA	TATCTGCACTGCCAAGACTGAGTG	CTTCTTGCTGGTCTTGCCATTCC	126	Langnaese et al. 2008

### Western blotting

Cells were lysed with RIPA buffer (VWR, USA) supplemented with protease inhibitors (Thermo Scientific). Lysates were centrifuged at 14,000 × g for 15 min at 4 °C, and protein concentrations were determined using the Bradford assay with BSA standards. Equal amounts of protein (50 µg) were separated by SDS-PAGE and transferred to PVDF membranes (Millipore) using the Trans-Blot^®^ Turbo™ system (Bio-Rad). Membranes were blocked with 3% skim milk for 1 h, incubated overnight at 4 °C with primary antibodies, and then incubated for 2 h at room temperature with HRP-conjugated secondary antibodies. Anti-XBP1 (Affinity Biosciences, Cat# AF5110, 1:1000), anti-CASP3 (Santa Cruz Biotechnology; Cat# sc-98785, 1:500), anti-CASP8 (Santa Cruz Biotechnology; Cat# sc-166320, 1:400), anti-CASP9 (Santa Cruz Biotechnology; Cat# sc-73548, 1:400), anti-CDH1 (St John’s Laboratory; Cat# STJ97370, 1:1000), anti-CDH2 (St John’s Laboratory; Cat# STJ94353, 1:1000) and housekeeping anti-GAPDH (Santa Cruz Biotechnology; Cat# sc-25778, 1:1000) were used as the primary antibodies. Goat anti-rabbit IgG (Santa Cruz Biotechnology, Cat# sc-2054, 1:5000) and goat anti-mouse IgG (Proteintech, Cat# SA00001-1, 1:5000) were used as secondary antibodies. Signals were detected using ECL substrate (Boster AR1170) and visualized on an Azure C280 imaging system. Band intensities were quantified by densitometric analysis using ImageJ software, and target protein expression levels were normalized to GAPDH.

### Colony formation assay

MCF7 and MDA-MB-231 cells were seeded at 10³ cells per well in 6-well plates and incubated at 37 °C in a CO_2_ incubator. Control and combination treatment groups were maintained for 10 days with medium replenishment every 48 h. At the end of the culture period, cells were washed with PBS and fixed in cold methanol at -20 °C for 10 min. Fixed colonies were stained with 5% crystal violet, and colony numbers were recorded.

### Statistical analysis

In this study, the Ct values of genes analyzed by qPCR were normalized using the 2^(*−ΔCt*)^ method with ACTB and CYPA as reference genes. Statistical comparisons of the groups were performed using SPSS 29.0. For multiple comparisons, one-way analysis of variance (ANOVA) was followed by Tukey’s post-hoc test, while pairwise comparisons were evaluated using Student’s *t-*test. A *p* ≤ 0.05 value was considered as statistically significant.

## Results

### Silencing of XBP1 gene and protein expression in MCF7 and MDA-MB-231 cells

**Fig. 1:**
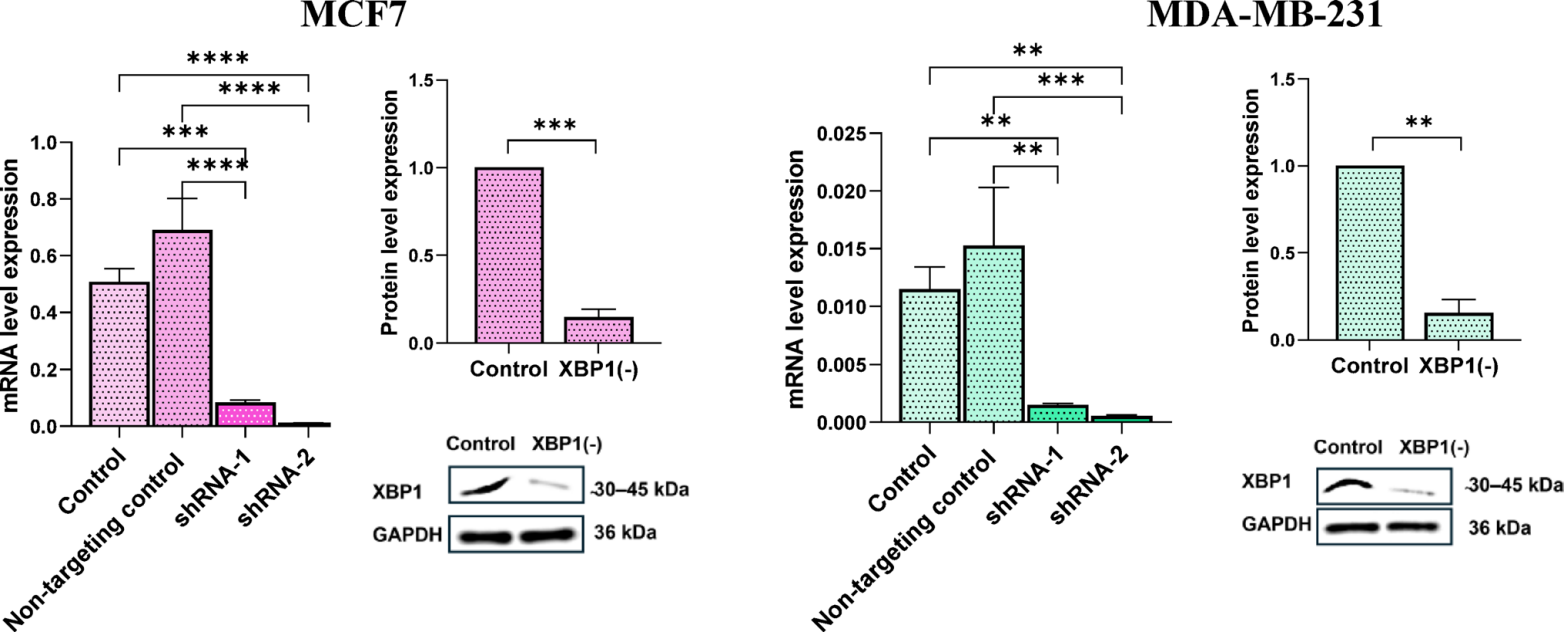
Efficiency of XBP1 gene silencing in MCF7 and MDA-MB-231 cells. XBP1 expression was evaluated in MCF7 and MDA-MB-231 cells at the mRNA and protein levels by qPCR and western blot analysis, respectively. Protein expression was assessed following shRNA-2-mediated silencing. Data are presented as mean ± SEM from three independent experiments (*n* = 3). Statistical analysis of qPCR data was performed using one-way ANOVA, whereas western blot data were analyzed using Student’s t-test. **p ≤ 0.01; ***p ≤ 0.001; ****p ≤ 0.0001

The shRNA constructs V2LHS_18216 (shRNA-1) and V3LHS_387388 (shRNA-2) are lentiviral vectors specifically designed to suppress XBP1 gene expression. To assess the silencing efficiency of XBP1, MCF7 and MDA-MB-231 cells were transduced with non-targeting control shRNA, shRNA-1, or shRNA-2. qPCR analysis showed that XBP1 expression was not significantly altered in the negative control group compared to untreated cells (*p* > 0.05). However, both shRNA-1 (*p* ≤ 0.001) and shRNA-2 (*p* ≤ 0.0001) significantly suppressed XBP1 expression in MCF7 cells and both shRNA-1 (*p* ≤ 0.01) and shRNA-2 (*p* ≤ 0.01) in MDA-MB-231 cells. shRNA-2 suppressed XBP1 expression ⁓40 fold in MCF7 cells and ⁓20.8 fold in MDA-MB-231 cells. (Fig. 1). These results indicate that shRNA-2 was more effective in silencing XBP1 in both cell lines; it was thereby selected for subsequent experiments.

To validate the XBP1 knockdown at the protein level, western blot analysis was performed on MCF7 and MDA-MB-231 cells transduced with shRNA-2. Compared to the control group, XBP1 protein expression was found to be reduced by approximately 10-fold in both cell lines (Fig. 1).

### Fluorescence microscopic examination of transduced cells

**Fig. 2:**
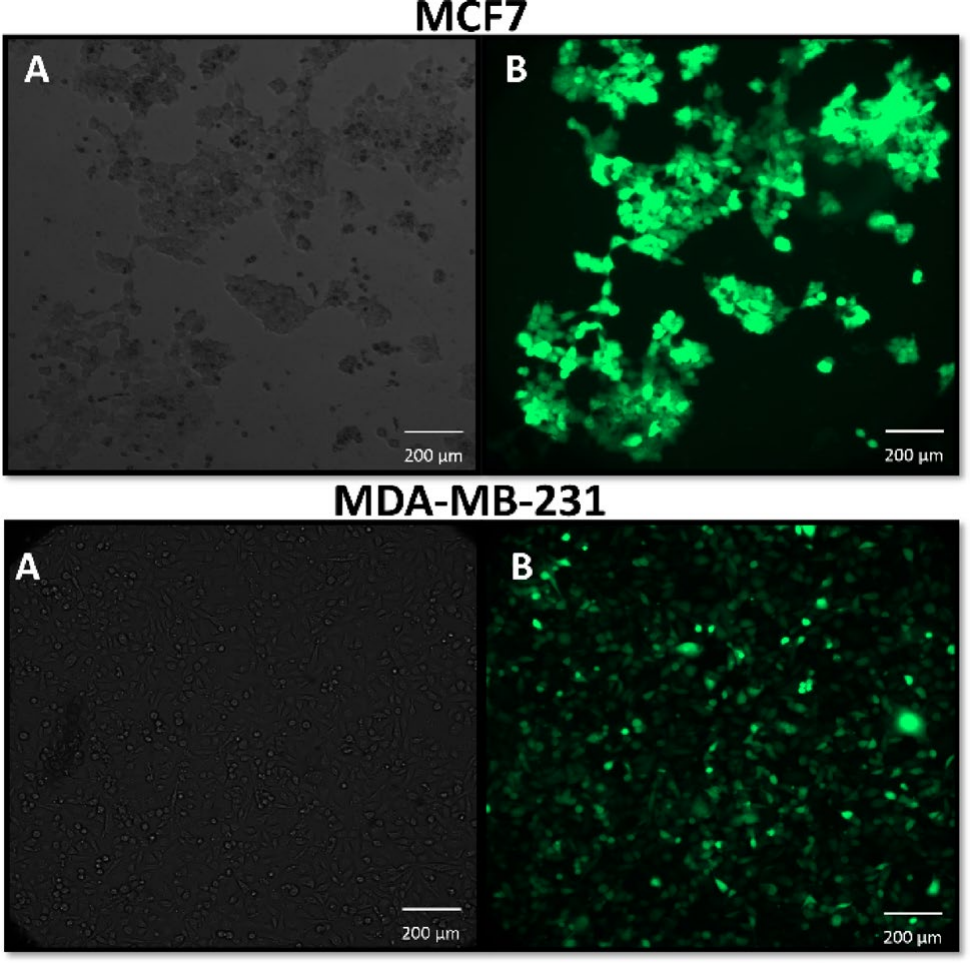
Representative microscopic images of transduced cells. Upper panels: MCF7; lower panels: MDA-MB-231. (**A**) Brightfield and (**B**) TurboGFP fluorescence images demonstrating transduction in the indicated cell lines. Images are representative of three independent experiments with similar results and were captured at 10X magnification, scale bars: 200 μm

MCF7 and MDA-MB-231 cells were transduced as described above. Transduction efficiency was assessed 48 h after transduction by monitoring TurboGFP reporter gene expression under a fluorescence microscope. The percentage of TurboGFP-positive cells and colonies was determined by manual counting under fluorescence microscopy. Based on these counts, the transduction units were calculated as 3.4 × 10^7^ TU/mL for MCF7 cells and 9.5 × 10^6^ TU/mL for MDA-MB-231 cells. Cells showing low TurboGFP expression (< 70%) were subsequently eliminated by approximately 10 days of puromycin selection. Representative fluorescence microscopy images of GFP-positive cells are shown in Fig. 2.

### Impact of Que and its combination with XBP1 silencing on breast cancer cell viability

**Fig. 3:**
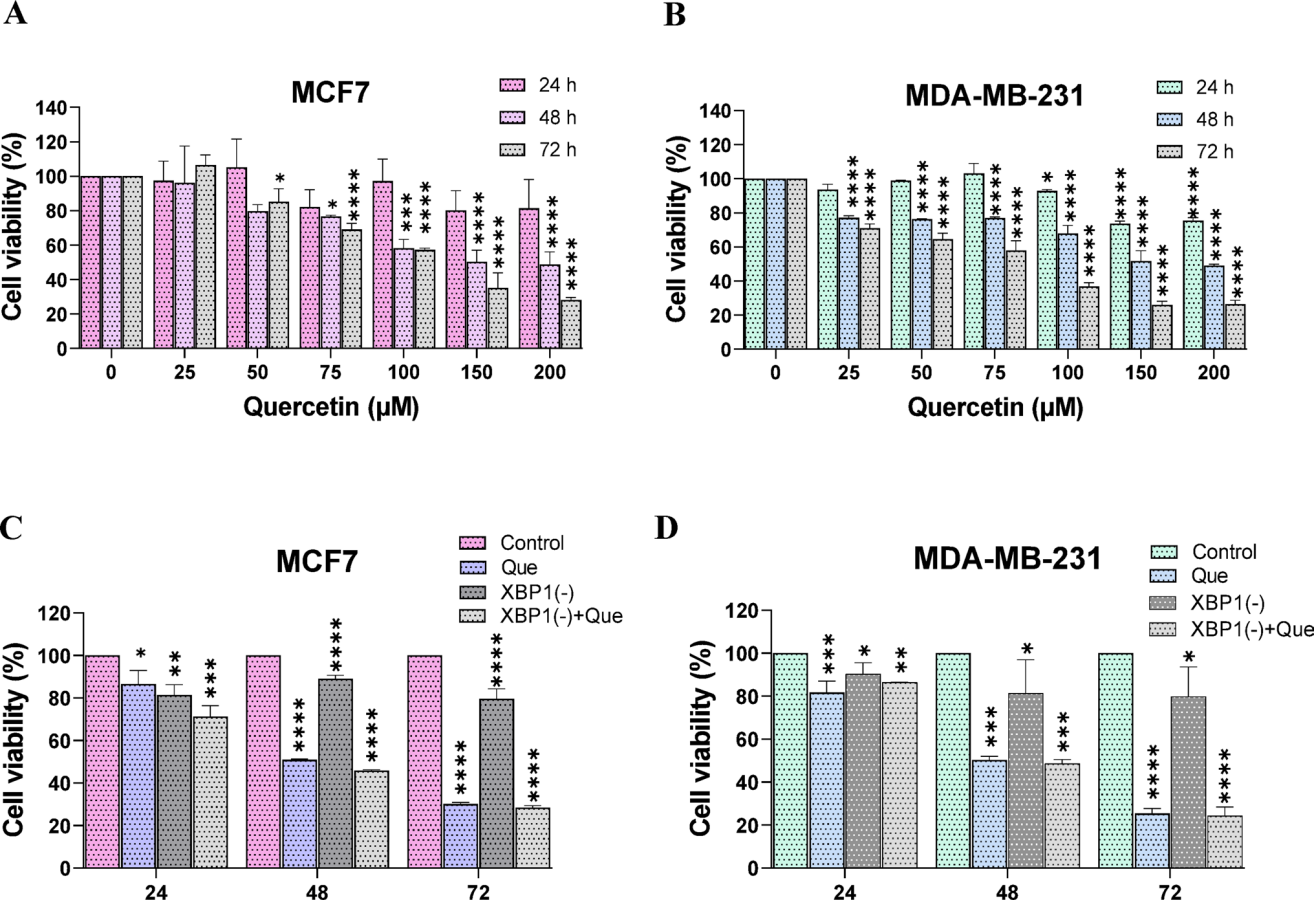
Que-induced cytotoxicity and the combined effects of Que and XBP1 silencing on breast cancer cells viability. (**A**, **B**) Cell viability of MCF7 and MDA-MB-231 cells after treatment with increasing concentrations of Que (0–200 µM) for 24, 48, and 72 h. (**C**) Cell viability of MCF7 cells in the Control, Que, XBP1(-), and XBP1(-) + Que groups at 24, 48, and 72 h, using Que at its IC_50_ concentration (154.262 µM). (**D**) Cell viability of MDA-MB-231 cells in the Control, Que, XBP1(-), and XBP1(-) + Que groups at 24, 48, and 72 h, using Que at its IC_50_ concentration (192.103 µM). Data are presented as mean ± SEM from three independent experiments (*n* = 3). Statistical analysis of cell viability was performed using one-way ANOVA followed by Tukey’s post-hoc test. *p ≤ 0.05; ** *p* ≤ 0.01; ***p ≤ 0.001; ****p ≤ 0.0001

The cytotoxic effects of different concentrations of Que, XBP1 gene silencing, and the combination of both treatments on MCF7 and MDA-MB-231 breast cancer cell lines were evaluated at 24th, 48th, and 72nd h using the XTT cell viability assay. This approach aimed to assess both the individual and synergistic impacts of the treatments on cell proliferation and viability over time. The IC_50_ values of Que were calculated as 154.262 µM (MCF7) and 192.103 µM (MDA-MB-231) for 48 h (Fig. 3). For subsequent experiments, Que was used at the IC_50_ determined from the cell viability assay.

### Que treatment, XBP1 silencing and combined treatment suppressed colony formation

**Fig. 4:**
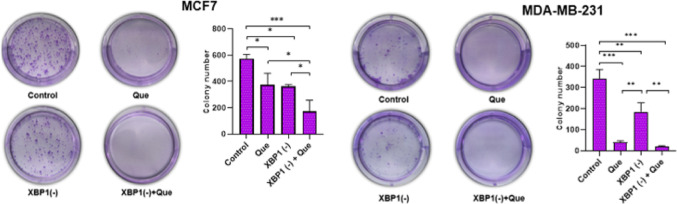
Colony formation capacity of MCF7 and MDA-MB-231 cells following Que treatment and XBP1 silencing. Representative colony images and quantitative colony counts are shown for MCF7 and MDA-MB-231 cells in the Control, Que, XBP1(-), and XBP1(-) + Que groups. Que was used at the IC_50_ concentration determined for each cell line. Colonies were counted manually. Data are presented as mean ± SEM from three independent experiments (*n* = 3). Statistical analysis was performed using one-way ANOVA followed by Tukey’s post-hoc test. *p ≤ 0.05; **p ≤ 0.01; ***p ≤ 0.001

Effects of Que treatment and XBP1 silencing, alone and in combination, on colony formation capacity of MCF7 and MDA-MB-231 cells were evaluated by colony formation assay. Que, XBP1 silencing, and their combined treatment significantly suppressed colony formation in both cell lines. In MCF7 cells, colony formation was inhibited by 35 ± 11.9% in the Que, 36 ± 1.4% in the XBP1(-), and 70 ± 13.2% in the combination groups compared to the control. Similarly, in MDA-MB-231 cells, the inhibition rates were 88 ± 0.6%, 46 ± 6%, and 94 ± 0.3%, respectively (Fig. 4).

### XBP1 silencing and Que combined treatment affected expressions of apoptosis associated genes in breast cancer cells

**Fig. 5:**
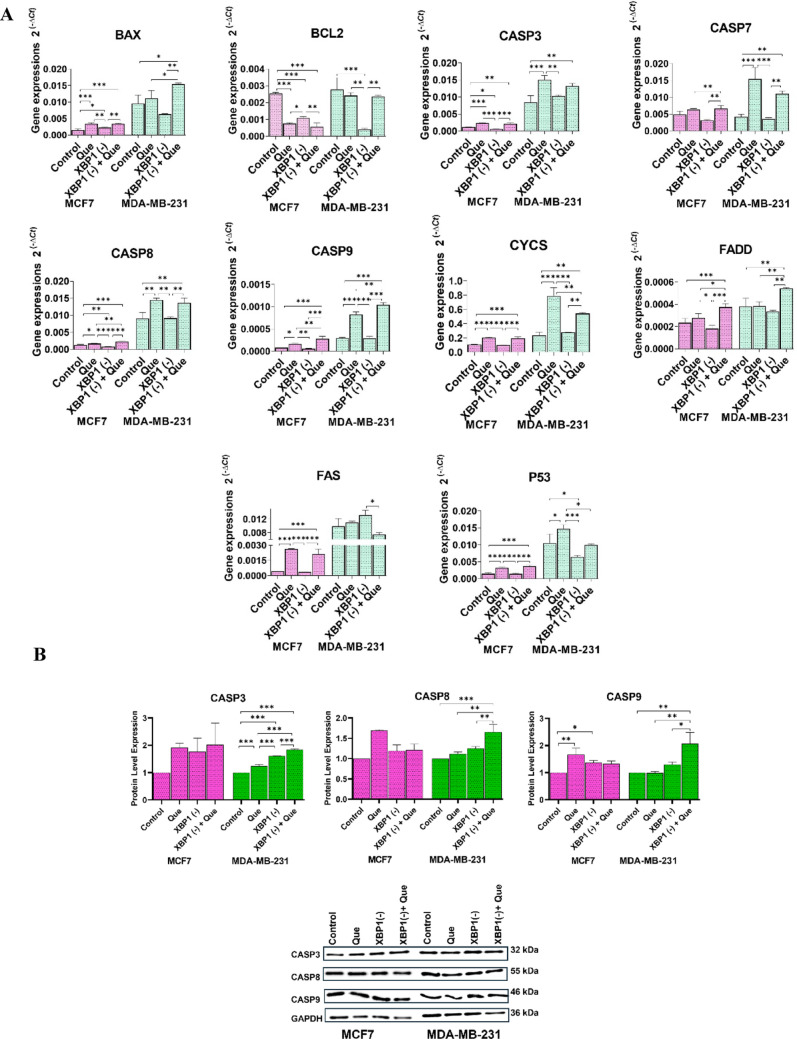
Effects of Que, XBP1 silencing, and their combination on expression levels of apoptosis-associated genes in MCF7 and MDA-MB-231 breast cancer cell lines. (**A**) Relative mRNA expression levels of BAX, BCL2, CASP3, CASP7, CASP8, CASP9, CYCS, and FADD were analyzed using qPCR in the Control, Que, XBP1(-), and XBP1(-) + Que groups. (**B**) Protein level expressions of CASP3, CASP8, and CASP9 were evaluated by western blot analysis in the same treatment groups. Bar graphs in panel B represent densitometric quantification of western blot bands performed using ImageJ and normalized to GAPDH. Que was applied at the IC_50_ concentration determined for each cell line. Data are presented as mean ± SEM from three independent experiments (*n* = 3). Statistical analysis was performed using one-way ANOVA. *p ≤ 0.05; **p ≤ 0.01; ***p ≤ 0.001

The effects of Que, XBP1 silencing, and their combination on the expression of key genes involved in apoptosis were evaluated in MCF7 and MDA-MB-231 breast cancer cells using qPCR and western blot analyses. In MCF7 cells, treatment with Que or the combination resulted in a marked upregulation of the pro-apoptotic gene BAX, accompanied by a strong downregulation of BCL2, suggesting a shift toward apoptotic signaling. Similarly, in MDA-MB-231 cells, BAX expression was increased following the combination treatment, while BCL2 expression was also reduced in the XBP1 knockdown group. Mitochondria-mediated apoptosis was further supported by the increased expression of cytochrome c (CYCS) in both cell lines following Que and combined treatments. This finding indicates enhanced release of mitochondrial apoptotic factors under these conditions. Also, an increased CASP3 expressions were observed in both cell lines upon treatment with Que and the combination. Protein expression levels were consistent with increased CASP3 levels. While CASP7 expression remained unchanged in MCF7 cells, it was elevated in MDA-MB-231 cells following Que and combined treatments. CASP8, a key initiator of the extrinsic apoptotic pathway, was upregulated at both mRNA and protein levels in both cell lines, particularly in response to Que and the combination. Expression and protein levels of CASP9, the initiator caspase of the intrinsic pathway, were also increased across all treatment groups in both cell lines. Among death receptor pathway regulators, FADD expression was notably upregulated only in the combination-treated groups in both cell lines. Likewise, FAS gene expression was elevated in MCF7 cells exposed to Que and the combination, while no significant change was observed in MDA-MB-231 cells. Expression of P53, a central tumor suppressor and regulator of apoptosis, was enhanced by Que and combination treatment in both MCF7 and MDA-MB-231 cells, highlighting its involvement in treatment-induced apoptotic responses. All together, these findings suggest that the combined inhibition of XBP1 and administration of Que effectively enhance both intrinsic and extrinsic apoptotic signaling in breast cancer cells, with more pronounced effects observed in the MCF7 cell line (Fig. 5).

### The combined treatment with XBP1 silencing and Que affected expressions of EMT and metastasis associated genes in breast cancer cells

**Fig. 6:**
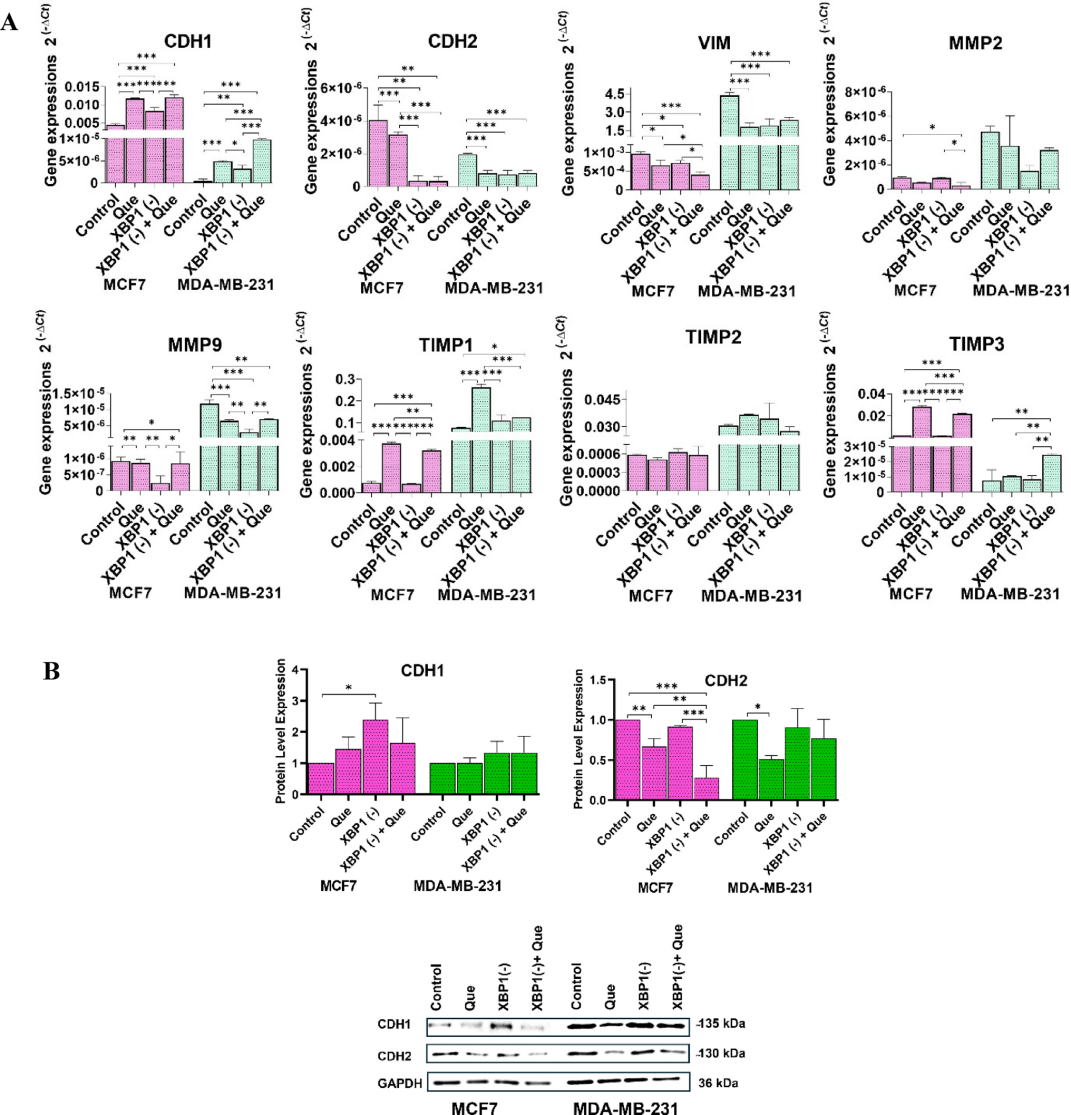
Effects of Que, XBP1 silencing, and their combination on EMT- and metastasis-associated genes in MCF7 and MDA-MB-231 breast cancer cell lines. (**A**) Relative mRNA expression levels of EMT/metastasis-associated markers were analyzed by qPCR in the Control, Que, XBP1(-), and XBP1(-) + Que groups. (**B**) Protein level expressions of CDH1 and CDH2 were evaluated by western blot analysis in the same treatment groups. Bar graphs in panel B represent densitometric quantification of western blot bands performed using ImageJ and normalized to GAPDH. Que was applied at the IC_50_ concentration determined for each cell line. Data are presented as mean ± SEM from three independent experiments (*n* = 3). Statistical analysis was performed using one-way ANOVA. *p ≤ 0.05; **p ≤ 0.01; ***p ≤ 0.001

The effects of Que, XBP1 silencing and their combination on the expression of key genes involved in EMT and metastasis were evaluated in MCF7 and MDA-MB-231 cells using qPCR and western blot analyses. CDH1 (E-cadherin), a tumor suppressor involved in maintaining epithelial integrity, was significantly upregulated in both cell lines following all treatments. In MCF7 cells, CDH1 mRNA expression were increased upon Que, XBP1(-), and combination treatments. CDH1 protein levels were increased with the highest fold-change observed in the XBP1(-) group. Similarly, CDH1 expression was elevated in MDA-MB-231 cells, especially in the XBP1(-) and combination groups. CDH2 (N-cadherin), a mesenchymal marker associated with invasion and metastasis, was markedly downregulated at the mRNA and protein levels in response to all treatments in both cell lines. These findings suggest a reversal cadherin switching, indicative of EMT inhibition. As an another mesenchymal marker, VIM (vimentin) expression was decreased in both cell lines, particularly in the combination group, supporting an anti-EMT effect. Regarding ECM remodeling, MMP2 expression was slightly reduced in MCF7 cells following combination treatment, while MMP9 expression was more strongly suppressed in both cell lines, particularly in the Que and combination groups. These reductions suggest inhibition of matrix degradation and metastatic potential. TIMP1, an inhibitor of MMPs, was upregulated in both cell lines after Que and combination treatments, while TIMP2 levels remained unchanged. TIMP3 expression was increased significantly in Que and combination groups of both MCF7 and MDA-MB-231 cells. These results indicate a shift toward ECM stabilization and reduced invasiveness. Collectively, these findings indicate that Que, particularly when combined with XBP1 knockdown, modulates EMT-related gene expression by enhancing epithelial markers and suppressing mesenchymal and matrix-degrading components (Fig. 6).

## Discussion

Cancer is a fatal disease characterized by uncontrolled cell proliferation. The inhibition of apoptosis contributes to tumor progression, whereas its induction is considered an effective therapeutic strategy. However, conventional cancer treatments often damage normal cells, leading to various adverse effects. In this regard, natural product derived compounds that selectively induce apoptosis in cancer cells present a promising alternative with fewer side effects. To survive under ER stress, cancer cells activate the UPR, which is also known to play a critical role in therapy resistance. Therefore, targeting UPR components to exacerbate ER stress and promote apoptosis has emerged as a potential anticancer strategy. In this context, the IRE1α-XBP1 signaling pathway functions as a key regulatory axis (Kim and Kim 2018). Notably, elevated XBP1 expression has been reported in various human malignancies, including breast cancer and hepatocellular carcinoma, suggesting its involvement in tumor progression and survival mechanisms (Koong et al. 2006). Fujimoto et al. (2003) demonstrated overexpression of XBP1 in tissue samples from 11 breast cancer patients who underwent surgical resection, as well as in five breast cancer cell lines (MDA-MB-453, CRL1500, YMB-1-E, MCF7 and HBL100). Their findings suggest that upregulation of the XBP1 gene plays a significant role in breast carcinogenesis.

In this study, the effects of Que and XBP1 gene silencing, and their combination were comprehensively investigated in MCF7 and MDA-MB-231 human breast cancer cell lines. Colony formation assays, a key in vitro method to assess clonal proliferation and long-term survival, revealed that both Que treatment and XBP1 silencing significantly reduced colony formation in both cell lines. Notably, the combination treatment led to a more pronounced decrease in colony numbers, indicating a substantial suppression of tumor cell proliferative capacity. Consistently, the combined treatment was also the most effective in reducing cell viability.

These findings support previous reports emphasizing the tumor-promoting and pro-angiogenic roles of XBP1. For instance, it was previously demonstrated that RNAi-mediated XBP1 knockdown suppressed tumor growth and enhanced chemosensitivity in murine breast cancer models (Zhang et al. 2020). Similarly, XBP1 silencing has been shown to enhance the antitumor activity of tumor-associated macrophages, suggesting potential effects on both tumor cells and the tumor microenvironment (Zhao et al. 2021).

In addition, Lin et al. (2023) identified XBP1 as a direct target of exosomal miR-3184-5p, which markedly inhibited proliferation, migration, and invasion while promoting apoptosis in gastric cancer cells. miR-3184-5p downregulated key oncogenic markers such as CD44, cyclin D1, MMP2, p65, p-AKT and p-STAT3, while upregulated ER stress-related markers including GRP78, IRE1, p-JNK and CHOP.

These findings are in line with accumulating evidence that XBP1 functions as a central regulator in cancer progression by interacting with multiple oncogenic pathways (Chen et al. 2020). Overexpression of XBP1 has been linked to endocrine resistance in breast cancer (Clarke and Cook 2015), and its cooperation with HIF1α has been implicated in angiogenesis and hypoxia-driven progression in triple-negative breast cancer (Chen et al. 2014). Consistently, XBP1 silencing under hypoxic conditions has been reported to reduce tumor growth and cell survival, highlighting XBP1 as a key survival factor under hypoxic stress (Romero-Ramirez et al. 2004). Persistent activation of the IRE1-XBP1 axis has been associated with tumor growth and metastasis in several malignancies, including breast, hepatocellular, pancreatic cancers and multiple myeloma. Furthermore, XBP1 has been linked to cancer cell differentiation, angiogenesis, susceptibility to oncoviral infections and EMT (Hu and Clarke 2019). Collectively, these findings support the hypothesis that targeting XBP1, especially in combination with natural compounds like Que, may provide a multifaceted approach to impair breast cancer progression. In the present study, given the central role of XBP1 in endoplasmic reticulum stress adaptation, the effects observed after XBP1 silencing, either alone or in combination with Que, may not be limited to the apoptosis-associated markers evaluated here. It is plausible that other branches of the unfolded protein response, particularly the PERK/eIF2α pathway, also contribute to the observed anti-proliferative and pro-apoptotic responses (Guichard et al. 2006; Zang et al. 2009). Activation of this axis has been associated with translational attenuation, stress adaptation and, under sustained stress conditions, apoptotic signaling (Hetz et al. 2020). Although these regulators were not examined at the protein level in the present study, their potential involvement should be considered in the interpretation of the present findings and addressed in future studies.

Gene and protein expression analyses of key caspases (CASP3, CASP7, CASP8 and CASP9), the principal mediators of apoptosis, confirmed that Que and XBP1 targeting effectively modulated apoptotic signaling. CASP3 and CASP7 act as executioner caspases, while CASP8 and CASP9 initiate the extrinsic (death receptor-mediated) and intrinsic (mitochondrial) apoptotic pathways, respectively (Fianco et al. 2018; Julien and Wells 2017). Natural compounds such as emodin, phenethyl isothiocyanate and fucosterol have been shown to induce apoptosis via CASP9 activation (Kim et al. 2015). In our study, combined treatment led to marked upregulation of CASP3, CASP8, and CASP9 gene expression in both cell lines, accompanied by corresponding increases in protein expression. The effects of Que, XBP1 silencing, and their combination on apoptosis-associated protein expression were further examined by western blotting in MCF7 and MDA-MB-231 cells. Densitometric quantification of CASP3, CASP8, and CASP9 bands normalized to GAPDH showed treatment-dependent alterations in protein levels across both cell lines. In general, the combined treatment resulted in stronger modulation of apoptosis-associated protein expression than either treatment alone, consistent with the pro-apoptotic effects observed in the other assays. Taken together, these findings support the involvement of apoptosis-related signaling and suggest that both intrinsic- and extrinsic pathway-associated markers may contribute to the observed response. In addition, the significant increase in CASP7 expression in MDA-MB-231 cells points to a possible cell line-specific apoptotic pattern, whereas elevated CASP8 levels may indicate a stronger contribution of extrinsic apoptosis-associated signaling in this cell line.

Expression analyses of BAX and BCL2 further supported the pro-apoptotic effects of the treatment. Increased BAX and reduced BCL2 expression were observed with Que and combination treatments, consistent with previous reports for MCF7 cells (Khorsandi et al. 2017). Since CYCS (cytochrome c) plays a central role in activating the caspase cascade through mitochondrial release, and BCL2 prevents this release to block apoptosis (He et al. 2021), the observed increase in CYCS expression upon combination treatment indicates activation of the intrinsic pathway. FAS and its ligand are constitutively expressed in various tissues and can be activated under appropriate stimulation (Makin and Hickman 2000). FAS-mediated apoptosis is a potential therapeutic target in some cancer types, and certain chemotherapeutic agents can upregulate FAS expression (Timmer et al. 2002). Absence of FADD expression has been associated with poor chemotherapy response and may serve as a prognostic factor (Tourneur and Chiocchia 2010). In our study, FAS expression was significantly increased in MCF7 cells following Que and combination treatments, while no significant change was observed in MDA-MB-231 cells. Interestingly, FADD expression significantly increased in MDA-MB-231 cells after combination treatment despite unchanged FAS levels, possibly indicating activation of extrinsic apoptosis through FAS-independent pathways such as TRAIL-R1/R2, which recruit FADD and form the death-inducing signaling complex (DISC; Schneider et al. 1997). P53, a tumor suppressor transcription factor, is activated in response to DNA damage, oncogenic stress, and other cellular insults. P53 inhibits tumor development by inducing cell cycle arrest, apoptosis and genomic stability, while also regulate DNA repair, metabolism, and antioxidant defense mechanisms (Chen 2016). In our study, P53 expression was significantly increased in MCF7 cells following combination treatment and in MDA-MB-231 cells with Que alone. Beyond its transcriptional role, P53 can directly promote mitochondrial apoptosis (Marchenko et al. 2000), supporting the idea that the combination treatment may enhance apoptosis via P53-mediated mechanisms.

The findings of the present study support the involvement of apoptosis through cell viability, colony formation, and apoptosis-associated gene/protein expression analyses. However, direct quantification of apoptotic cell populations was not performed. Therefore, techniques such as Annexin V/PI flow cytometry or live-dead assays would be valuable in future studies to more precisely determine the proportion of apoptotic cells following Que treatment, XBP1 silencing, and their combination. Inclusion of such assays would further strengthen the mechanistic interpretation of the pro-apoptotic effects observed here.

EMT is a crucial mechanism in cancer progression, particularly in invasion and metastasis. During EMT, epithelial cells lose polarity and adhesion, acquiring mesenchymal traits that enhance mobility and invasiveness. This transition involves downregulation of epithelial markers like CDH1 and upregulation of mesenchymal markers such as CDH2 and VIM (Kalluri and Weinberg 2009). Overexpression of N-cadherin (CDH2) has been associated with migration, angiogenesis, aggressiveness and metastasis in multiple cancers and is elevated in patient serum samples compared to healthy controls (Assidi 2022). Beyond the pro-apoptotic effects observed in the present study, our findings also suggest that Que and XBP1 silencing may influence EMT-associated regulation in breast cancer cells. In our study, Que, XBP1 silencing, and particularly the combined treatment increased CDH1 expression while decreasing CDH2 and VIM expression in both cell lines. This effect was more pronounced in MCF7 cells, suggesting a treatment-associated shift in EMT-related molecular characteristics that may be consistent with partial reversal of the mesenchymal phenotype. Notably, even in the triple-negative MDA-MB-231 cell line, the observed upregulation of CDH1 together with suppression of CDH2 and VIM suggests that this therapeutic approach may also be relevant in more aggressive breast cancer subtypes. Given that EMT is closely associated with invasion, metastasis, and therapeutic resistance, these molecular changes may be biologically meaningful (Hashemi et al. 2022). In agreement with our findings, previous studies have reported that Que upregulates E-cadherin and suppresses vimentin in TNBC cells, thereby reducing motility and metastatic potential (Srinivasan et al. 2016). However, since the present study did not include functional assays such as migration or invasion analyses, these findings should be interpreted as molecular evidence of EMT-associated modulation rather than definitive proof of reduced metastatic behavior.

MMP2 and MMP9, key matrix metalloproteinases involved in ECM degradation, facilitate invasion and early metastasis by breaking down the basement membrane (Jiang and Li 2021). Their overexpression is associated with poor prognosis in several cancers, including retinoblastoma, bladder, oral and ovarian epithelial cancers (Deng et al. 2019; Jia et al. 2017; Miao et al. 2017; Zhu et al. 2019). In our study, MMP9 expression was significantly reduced by combination treatment in both cell lines, while MMP2 suppression was observed only in MCF7 cells. These changes suggest that Que and XBP1 silencing may impair metastatic processes. Similarly, Balakrishnan et al. (2016) reported that Que-loaded nanoparticles inhibited MMP2/9 activity and invasion in breast cancer models. MMP activity is regulated not only at the transcriptional level but also through tissue inhibitors of metalloproteinases (TIMPs). The TIMP family (TIMP1-4) directly binds and inhibits MMPs, preventing excessive ECM degradation (Nagase et al. 2006). In our study, TIMP1 expression was significantly increased by Que and combination treatments in both cell lines. While TIMP1 is known for its MMP-inhibitory role, it can also promote cell survival and proliferation through noncanonical pathways involving CD63 and PI3K/AKT signaling (Eckfeld et al. 2019). Therefore, its functional impact may vary depending on cellular context. In light of the concurrent reduction in MMP9 expression, TIMP1 upregulation in our study likely reflects an anti-invasive effect. TIMP2 expression remained unchanged in both cell lines, suggesting a limited or nonresponsive role to the applied treatments. This aligns with previous findings that reported inconsistent associations between TIMP2 expression and metastatic potential (Jackson et al. 2017). Conversely, TIMP3 expression was significantly elevated in MCF7 cells with Que and combination treatments, and in MDA-MB-231 cells only after the combined treatment. TIMP3 is well-documented to promote apoptosis and inhibit angiogenesis, supporting its tumor-suppressive role (Eckfeld et al. 2019). The selective upregulation of TIMP3 in TNBC cells under combination treatment suggests that the dual targeting of XBP1 and Que may synergistically inhibit metastasis through multiple regulatory axes.

In addition to its established roles in ER stress adaptation and tumor progression, XBP1 has also been implicated in the regulation of lipid and glucose metabolism (Piperi et al. 2016). Therefore, the effects of quercetin and XBP1 silencing observed in the present study may extend beyond apoptosis- and EMT-associated pathways and may also involve metabolic regulators. In particular, SREBP-associated lipid regulatory signaling and FoxO-related glucose metabolism pathways may represent relevant mechanistic axes, since both are functionally linked to cellular metabolic adaptation (Ning et al. 2011; Zhou et al. 2011). In this context, the combined effects of Que and XBP1 silencing may influence metabolic homeostasis in breast cancer cells in addition to suppressing proliferation and modulating apoptosis-associated responses. However, because metabolic markers were not directly evaluated in the present study, this possibility remains speculative and should be investigated in future studies using selected lipid- and glucose-metabolism-related markers.

**Fig. 7:**
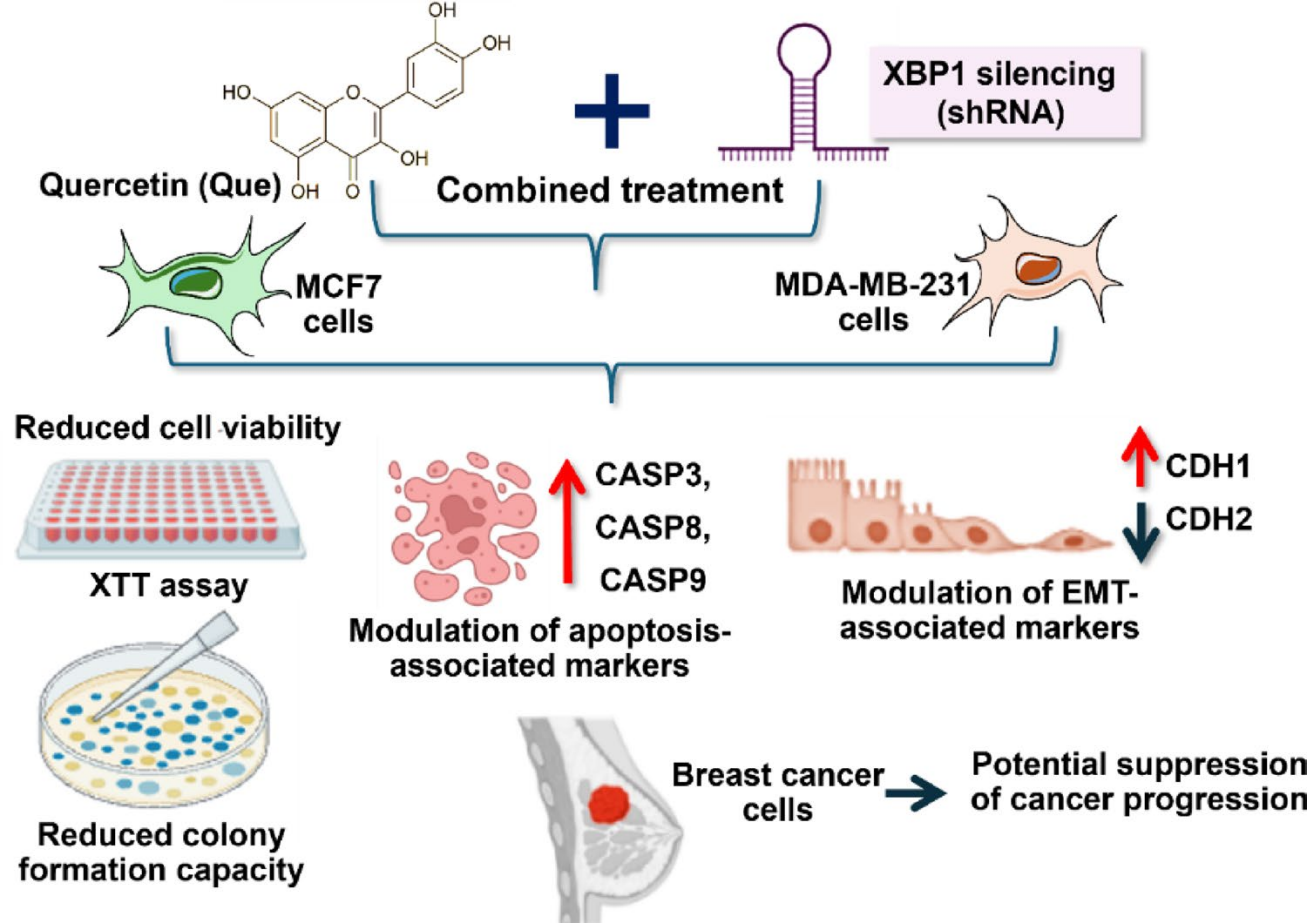
Proposed mechanistic overview of the combined treatment with Que and XBP1 silencing in MCF7 and MDA-MB-231 breast cancer cells. The schematic summarizes the treatment-associated reduction in cell viability, suppression of colony formation, modulation of apoptosis-associated markers, and EMT-related molecular changes observed in the present study. The relationships shown are presented as a conceptual summary of the current findings

A conceptual summary of the combined treatment effects is presented in Fig. 7. As illustrated, the combination of quercetin and XBP1 silencing was associated with reduced cell viability, decreased colony formation capacity, modulation of apoptosis-associated markers, and EMT-related molecular changes in both breast cancer cell lines.

## Conclusion

This study demonstrates that shRNA-mediated silencing of XBP1, Que treatment and their combination, exert significant anti-cancer effects on MCF7 and MDA-MB-231 human breast cancer cell lines. The combined application notably suppressed cell proliferation and colony formation while promoting apoptosis, as evidenced by the upregulation of CASP3, CASP8 and CASP9 at both mRNA and protein levels. Although the observed alterations in apoptosis-related genes support the involvement of apoptotic signaling, additional protein-level validation such as cleaved PARP and necroptosis-associated markers would further refine the mechanistic distinction between apoptosis and alternative cell death pathways (Bertheloot et al. 2021; Chaitanya et al. 2010). Moreover, alterations in EMT- and metastasis-related gene expression particularly the increase in CDH1 and decrease in CDH2 suggest a reduction in the invasive potential of cancer cells. These findings indicate that targeting XBP1 in conjunction with Que treatment may represent a promising therapeutic strategy for breast cancer, warranting further investigation through 3D culture systems and in vivo models.

## References

[CR1] Aalinkeel R, Bindukumar B, Reynolds JL, Sykes DE, Mahajan SD, Chadha KC, Schwartz SA (2008) The dietary bioflavonoid, quercetin, selectively induces apoptosis of prostate cancer cells by down-regulating the expression of heat shock protein 90. Prostate 68:1773–1789. 10.1002/pros.20845 18726985 10.1002/pros.20845 PMC2826114

[CR2] Assidi M (2022) High N-cadherin protein expression in ovarian cancer predicts poor survival and triggers cell invasion. Front Oncol 12:870820. 10.3389/fonc.2022.870820 35574323 10.3389/fonc.2022.870820 PMC9096138

[CR3] Bakir B, Chiarella AM, Pitarresi JR, Rustgi AK (2020) EMT, MET, plasticity, and tumor metastasis. Trends Cell Biol 30:764–776. 10.1016/j.tcb.2020.07.003 32800658 10.1016/j.tcb.2020.07.003 PMC7647095

[CR4] Balakrishnan S, Bhat FA, Raja Singh P, Mukherjee S, Elumalai P, Das S, Patra CR, Arunakaran J (2016) Gold nanoparticle-conjugated quercetin inhibits epithelial–mesenchymal transition, angiogenesis and invasiveness via EGFR/VEGFR-2-mediated pathway in breast cancer. Cell Prolif 49:678–697. 10.1111/cpr.12296 27641938 10.1111/cpr.12296 PMC6496551

[CR5] Barzaman K, Karami J, Zarei Z, Hosseinzadeh A, Kazemi MH, Moradi-Kalbolandi S, Safari E, Farahmand L (2020) Breast cancer: Biology, biomarkers, and treatments. Int Immunopharmacol 84:106535. 10.1016/j.intimp.2020.106535 32361569 10.1016/j.intimp.2020.106535

[CR6] Bertheloot D, Latz E, Franklin BS (2021) Necroptosis, pyroptosis and apoptosis: an intricate game of cell death. Cell Mol Immunol 18(5):1106–1121. 10.1038/s41423-020-00630-3 33785842 10.1038/s41423-020-00630-3 PMC8008022

[CR7] Bianchini G, Balko JM, Mayer IA, Sanders ME, Gianni L (2016) Triple-negative breast cancer: challenges and opportunities of a heterogeneous disease. Nat Rev Clin Oncol 13:674–690. 10.1038/nrclinonc.2016.66 27184417 10.1038/nrclinonc.2016.66 PMC5461122

[CR8] Chaitanya GV, Steven AJ, Babu PP (2010) PARP-1 cleavage fragments: Signatures of cell-death proteases in neurodegeneration. Cell Commun Signal 22:8:31. 10.1186/1478-811X-8-31 10.1186/1478-811X-8-31 PMC3022541 21176168

[CR9] Chao YL, Shepard CR, Wells A (2010) Breast carcinoma cells re-express E-cadherin during mesenchymal to epithelial reverting transition. Mol Cancer 9:179. 10.1186/1476-4598-9-179 20609236 10.1186/1476-4598-9-179 PMC2907333

[CR10] Chen J (2016) The cell-cycle arrest and apoptotic functions of p53 in tumor initiation and progression. Cold Spring Harb Perspect Med 6:a026104. 10.1101/cshperspect.a026104 26931810 10.1101/cshperspect.a026104 PMC4772082

[CR12] Chen X, Iliopoulos D, Zhang Q, Tang Q, Greenblatt MB et al (2014) XBP1 promotes triple-negative breast cancer by controlling the HIF1α pathway. Nature 508:103–107. 10.1038/nature13028 24670641 10.1038/nature13119 PMC4105133

[CR11] Chen S, Chen J, Hua X, Sun Y, Cui R, Sha J, Zhu X (2020) The emerging role of XBP1 in cancer. Biomed Pharmacother 127:110069. 10.1016/j.biopha.2020.110069 32294597 10.1016/j.biopha.2020.110069

[CR13] Clarke R, Cook KL (2015) Unfolding the role of stress response signaling in endocrine resistant breast cancers. Front Oncol 5:140. 10.3389/fonc.2015.00140 26157705 10.3389/fonc.2015.00140 PMC4475795

[CR14] Cuevas EP, Eraso P, Mazón MJ, Santos V, Moreno-Bueno G, Cano A, Portillo F (2017) LOXL2 drives epithelial–mesenchymal transition via activation of IRE1–XBP1 signalling pathway. Sci Rep 7:44988. 10.1038/srep44988 28332555 10.1038/srep44988 PMC5362953

[CR15] Deepika, Maurya PK (2022) Health benefits of quercetin in age-related diseases. Molecules 27:2498. 10.3390/molecules27082498 35458696 10.3390/molecules27082498 PMC9032170

[CR16] Deng W, Peng W, Wang T, Chen J, Zhu S (2019) Overexpression of MMPs functions as a prognostic biomarker for oral cancer patients: A systematic review and meta-analysis. Oral Health Prev Dent 17:505–514. 10.3290/j.ohpd.a43636 31825023 10.3290/j.ohpd.a43636

[CR17] Eckfeld C, Häußler D, Schoeps B, Hermann CD, Krüger A (2019) Functional disparities within the TIMP family in cancer: Hints from molecular divergence. Cancer Metastasis Rev 38:469–481. 10.1007/s10555-019-09812-6 31529339 10.1007/s10555-019-09812-6

[CR18] Erlund I (2004) Review of the flavonoids quercetin, hesperetin, and naringenin. Dietary sources, bioactivities, bioavailability, and epidemiology. Nutr Res 24:851–874. 10.1016/j.nutres.2004.07.005

[CR20] Eroğlu C, Avcı E, Vural H, Kurar E (2018) Anticancer mechanism of Sinapic acid in PC-3 and LNCaP human prostate cancer cell lines. Gene 671:127–134. 10.1016/j.gene.2018.05.049 29792952 10.1016/j.gene.2018.05.049

[CR19] Eroglu Gunes C (2023) Boric acid shows ER stress and apoptosis mediated anticancer activity in human pancreatic cancer MIA PaCa-2 and PANC-1 cells. Selcuk Med J 39:1–6. 10.30733/std.2023.01608

[CR21] Eroğlu Güneş C, Güçlü E, Vural H, Kurar E (2021) Knockdown of lncRNA ZEB2NAT suppresses epithelial mesenchymal transition, metastasis and proliferation in breast cancer cells. Gene 805:145904. 10.1016/j.gene.2021.145904 34418470 10.1016/j.gene.2021.145904

[CR22] Fianco G, Contadini C, Ferri A, Cirotti C, Stagni V, Barilà D (2018) Caspase-8: A novel target to overcome resistance to chemotherapy in glioblastoma. Int J Mol Sci 19:3798. 10.3390/ijms19123798 30501030 10.3390/ijms19123798 PMC6320982

[CR23] Fujimoto T, Onda M, Nagai H, Nagahata T, Ogawa K, Emi M (2003) Upregulation and overexpression of human X-box binding protein 1 (hXBP-1) gene in primary breast cancers. Breast Cancer 10:301–306. 10.1007/BF02967649 14634507 10.1007/BF02967649

[CR24] Guichard C, Pedruzzi E, Fay M, Marie JC, Braut-Boucher F et al (2006) Dihydroxyphenylethanol induces apoptosis by activating serine/threonine protein phosphatase PP2A and promotes the endoplasmic reticulum stress response in human colon carcinoma cells. Carcinogenesis 27(9):1812–1827. 10.1093/carcin/bgl009 16524888 10.1093/carcin/bgl009

[CR25] Hanasaki Y, Ogawa S, Fukui S (1994) The correlation between active oxygens scavenging and antioxidative effects of flavonoids. Free Radic Biol Med 16:845–850. 10.1016/0891-5849(94)90192-9 8070690 10.1016/0891-5849(94)90202-x

[CR26] Harborne JB, Williams CA (2000) Advances in flavonoid research since 1992. Phytochemistry 55:481–504. 10.1016/S0031-9422(00)00235-1 11130659 10.1016/s0031-9422(00)00235-1

[CR27] Harnoss JM, Le-Thomas A, Reichelt M, Guttman O, Wu TD et al (2020) IRE1α disruption in triple-negative breast cancer cooperates with antiangiogenic therapy by reversing ER stress adaptation and remodeling the tumor microenvironment. Cancer Res 80:2368–2379. 10.1158/0008-5472.CAN-19-3108 32265225 10.1158/0008-5472.CAN-19-3108 PMC7272310

[CR28] Hashemi M, Arani HZ, Orouei S, Fallah S, Ghorbani A et al (2022) EMT mechanism in breast cancer metastasis and drug resistance: Revisiting molecular interactions and biological functions. Biomed Pharmacother 155:113774. 10.1016/j.biopha.2022.113774 36271556 10.1016/j.biopha.2022.113774

[CR29] He Y, Zhao T, Chen F, Song C, Zhong C, Luo Z (2021) Functional analysis of the promoter regions of two apoptosis-related genes (Bcl-2 and Cycs) and their regulation by Zn in yellow catfish. Int J Mol Sci 22:6291. 10.3390/ijms22126291 34208159 10.3390/ijms22126291 PMC8230946

[CR30] Hetz C, Zhang K, Kaufman RJ (2020) Mechanisms, regulation and functions of the unfolded protein response. Nat Rev Mol Cell Biol 21(8):421–438. 10.1038/s41580-020-0250-z 32457508 10.1038/s41580-020-0250-z PMC8867924

[CR31] Hu R, Clarke R (2019) Roles of Spliced and Unspliced XBP1 in Breast Cancer. In: Clarke R (ed) The Unfolded Protein Response in Cancer. Cancer Drug Discovery and Development. Humana, Cham. 10.1007/978-3-030-05067-2_6

[CR32] Hu R, Warri A, Jin L, Zwart A, Riggins RB, Fang HB, Clarke R (2015) NF-κB signaling is required for XBP1 (unspliced and spliced)-mediated effects on antiestrogen responsiveness and cell fate decisions in breast cancer. Mol Cell Biol 35:379–390. 10.1128/MCB.00847-14 25368386 10.1128/MCB.00847-14 PMC4272419

[CR33] Huang Y, Hong W, Wei X (2022) The molecular mechanisms and therapeutic strategies of EMT in tumor progression and metastasis. J Hematol Oncol 15:129. 10.1186/s13045-022-01347-8 36076302 10.1186/s13045-022-01347-8 PMC9461252

[CR34] Jackson HW, Defamie V, Waterhouse P, Khokha R (2017) TIMPs: versatile extracellular regulators in cancer. Nat Rev Cancer 7:38–53. 10.1038/nrc.2016.115 10.1038/nrc.2016.115 27932800

[CR35] Jia H, Zhang Q, Liu F, Zhou D (2017) Prognostic value of MMP-2 for patients with ovarian epithelial carcinoma: a systematic review and meta-analysis. Arch Gynecol Obstet 295:689–696. 10.1007/s00404-016-4257-9 27995372 10.1007/s00404-016-4257-9

[CR36] Jiang H, Li H (2021) Prognostic values of tumoral MMP2 and MMP9 overexpression in breast cancer: a systematic review and meta-analysis. BMC Cancer 21:149. 10.1186/s12885-021-07860-2 33568081 10.1186/s12885-021-07860-2 PMC7877076

[CR37] Julien O, Wells JA (2017) Caspases and their substrates. Cell Death Differ 24:1380–1389. 10.1038/cdd.2017.44 28498362 10.1038/cdd.2017.44 PMC5520456

[CR38] Kalluri R, Weinberg RA (2009) The basics of epithelial-mesenchymal transition. J Clin Invest 119:1420–1428. 10.1172/JCI39104 19487818 10.1172/JCI39104 PMC2689101

[CR39] Kashyap D, Garg VK, Goel N (2021) Intrinsic and extrinsic pathways of apoptosis: Role in cancer development and prognosis. Adv Protein Chem Struct Biol 125:73–120. 10.1016/bs.apcsb.2021.01.003 33931145 10.1016/bs.apcsb.2021.01.003

[CR40] Khorsandi L, Orazizadeh M, Niazvand F, Abbaspour MR, Mansouri E et al (2017) Quercetin induces apoptosis and necroptosis in MCF7 breast cancer cells. Bratisl Lek Listy 118:123–128. 10.4149/BLL_2017_025 28814095 10.4149/BLL_2017_025

[CR42] Kim C, Kim B (2018) Anti-cancer natural products and their bioactive compounds inducing ER stress-mediated apoptosis: A review. Nutrients 10:1021. 10.3390/nu10081021 30081573 10.3390/nu10081021 PMC6115829

[CR41] Kim B, Srivastava SK, Kim SH (2015) Caspase-9 as a therapeutic target for treating cancer. Expert Opin Ther Targets 19:113–127. 10.1517/14728222.2014.961425 25256701 10.1517/14728222.2014.961425

[CR43] Koong AC, Chauhan V, Romero-Ramirez L (2006) Targeting XBP-1 as a novel anti-cancer strategy. Cancer Biol Ther 5:756–759. 10.4161/cbt.5.7.2973 16861911 10.4161/cbt.5.7.2973

[CR44] Korsmeyer SJ, Shutter JR, Veis DJ, Merry DE, Oltvai ZN (1993) Bcl-2/Bax: A rheostat that regulates an anti-oxidant pathway and cell death. Semin Cancer Biol 4:327–332 8142617

[CR45] Langnaese K, John R, Schweizer H, Ebmeyer U, Keilhoff G (2008) Selection of reference genes for quantitative real-time PCR in a rat asphyxial cardiac arrest model. BMC Mol Biol 9:53. 10.1186/1471-2199-9-53 18505597 10.1186/1471-2199-9-53 PMC2430208

[CR46] Leung-Hagesteijn C, Erdmann N, Cheung G, Keats JJ, Stewart AK, Reece DE, Chung KC, Tiedemann RE (2013) Xbp1s-negative tumor B cells and pre-plasmablasts mediate therapeutic proteasome inhibitor resistance in multiple myeloma. Cancer Cell 24:289–304. 10.1016/j.ccr.2013.08.009 24029229 10.1016/j.ccr.2013.08.009 PMC4118579

[CR48] Li JJ, Wang JJ, Yu Q, Wang M, Zhang SX (2009) Endoplasmic reticulum stress is implicated in retinal inflammation and diabetic retinopathy. FEBS Lett 583:1521–1527. 10.1016/j.febslet.2009.04.007 19364508 10.1016/j.febslet.2009.04.007 PMC2691649

[CR47] Li H, Chen X, Gao Y, Wu J, Zeng F, Song F (2015) XBP1 induces snail expression to promote epithelial-to-mesenchymal transition and invasion of breast cancer cells. Cell Signal 27:82–89. 10.1016/j.cellsig.2014.09.018 25280941 10.1016/j.cellsig.2014.09.018

[CR49] Lin S, Que Y, Que C, Li F, Deng M, Xu D (2023) Exosome miR-3184-5p inhibits gastric cancer growth by targeting XBP1 to regulate the AKT, STAT3, and IRE1 signalling pathways. Asia Pac J Clin Oncol 19:e27–e38. 10.1111/ajco.13663 35394683 10.1111/ajco.13663

[CR50] Liou HC, Boothby MR, Finn PW, Davidon R, Nabavi N, Zeleznik-Le NJ, Ting JP, Glimcher LH (1990) A new member of the leucine zipper class of proteins that binds to the HLA DR alpha promoter. Science 247:1581–1584. 10.1126/science.2321018 2321018 10.1126/science.2321018

[CR51] Ma JH, Wang JJ, Zhang SX (2014) The unfolded protein response and diabetic retinopathy. J Diabetes Res 2014:160140. 10.1155/2014/160140 25530974 10.1155/2014/160140 PMC4229964

[CR52] Makin G, Hickman JA (2000) Apoptosis and cancer chemotherapy. Cell Tissue Res 301:143–152. 10.1007/s004419900160 10928287 10.1007/s004419900160

[CR53] Marchenko ND, Zaika A, Moll UM (2000) Death signal-induced localization of p53 protein to mitochondria. A potential role in apoptotic signaling. J Biol Chem 275:16202–16212. 10.1074/jbc.275.21.16202 10821866 10.1074/jbc.275.21.16202

[CR54] Miao C, Liang C, Zhu J, Xu A, Zhao K et al (2017) Prognostic role of matrix metalloproteinases in bladder carcinoma: a systematic review and meta-analysis. Oncotarget 8:32309–32321. 10.18632/oncotarget.15907 28427222 10.18632/oncotarget.15907 PMC5458286

[CR55] Middleton EJ, Kandaswami C, Theoharides TC (2000) The effects of plant flavonoids on mammalian cells: implications for inflammation, heart disease, and cancer. Pharmacol Rev 52:673–751 11121513

[CR56] Mohammad RM, Muqbil I, Lowe L, Yedjou C, Hsu HY et al (2015) Broad targeting of resistance to apoptosis in cancer. Semin Cancer Biol 35 Suppl:S78–103. 10.1016/j.semcancer.2015.03.001 25936818 10.1016/j.semcancer.2015.03.001 PMC4720504

[CR57] Murota K, Terao J (2003) Antioxidative flavonoid quercetin: Implication of its intestinal absorption and metabolism. Arch Biochem Biophys 417:12–17. 10.1016/s0003-9861(03)00284-4 12921774 10.1016/s0003-9861(03)00284-4

[CR58] Nagase H, Visse R, Murphy G (2006) Structure and function of matrix metalloproteinases and TIMPs. Cardiovasc Res 69:562–573. 10.1016/j.cardiores.2005.12.002 16405877 10.1016/j.cardiores.2005.12.002

[CR59] Ning J, Hong T, Ward A, Pi J, Liu Z et al (2011) Constitutive role for IRE1α-XBP1 signaling pathway in the insulin-mediated hepatic lipogenic program. Endocrinology 152(6):2247–2255. 10.1210/en.2010-1036 21447637 10.1210/en.2010-1036 PMC3100623

[CR60] Park M, Kim D, Ko S, Kim A, Mo K, Yoon H (2022) Breast cancer metastasis: Mechanisms and therapeutic implications. Int J Mol Sci 23:6806. 10.3390/ijms23126806 35743249 10.3390/ijms23126806 PMC9224686

[CR61] Piperi C, Adamopoulos C, Papavassiliou AG (2016) XBP1: A Pivotal transcriptional regulator of glucose and lipid metabolism. Trends Endocrinol Metab 27(3):119–122. 10.1016/j.tem.2016.01.001 26803729 10.1016/j.tem.2016.01.001

[CR62] Romero-Ramirez L, Cao H, Nelson D, Hammond E, Lee AH et al (2004) XBP1 is essential for survival under hypoxic conditions and is required for tumor growth. Cancer Res 64:5943–5947. 10.1158/0008-5472.CAN-04-1606 15342372 10.1158/0008-5472.CAN-04-1606

[CR63] Ryoo HD, Domingos PM, Kang MJ, Steller H (2007) Unfolded protein response in a Drosophila model for retinal degeneration. EMBO J 26:242–252. 10.1038/sj.emboj.7601477 17170705 10.1038/sj.emboj.7601477 PMC1782370

[CR64] Sak K (2014) Cytotoxicity of dietary flavonoids on different human cancer types. Pharmacogn Rev 8:122–146. 10.4103/0973-7847.134247 25125885 10.4103/0973-7847.134247 PMC4127821

[CR65] Schneider P, Thome M, Burns K, Bodmer JL, Hofmann K, Kataoka T, Holler N, Tschopp J (1997) TRAIL receptors 1 (DR4) and 2 (DR5) signal FADD-dependent apoptosis and activate NF-kappaB. Immunity 7:831–836. 10.1016/s1074-7613(00)80401-x 9430228 10.1016/s1074-7613(00)80401-x

[CR67] Siegel RL, Miller KD, Wagle NS, Jemal A (2023) Cancer statistics, 2023. CA Cancer J Clin 73:17–48. 10.3322/caac.21763 36633525 10.3322/caac.21763

[CR66] Siegel RL, Kratzer TB, Giaquinto AN, Sung H, Jemal A (2025) Cancer statistics, 2025. CA Cancer J Clin 75:10–45. 10.3322/caac.21871 39817679 10.3322/caac.21871 PMC11745215

[CR68] Singh P, Arif Y, Bajguz A, Hayat S (2021) The role of quercetin in plants. Plant Physiol Biochem 166:10–19. 10.1016/j.plaphy.2021.05.023 34087741 10.1016/j.plaphy.2021.05.023

[CR69] Srinivasan A, Thangavel C, Liu Y, Shoyele S, Den RB, Selvakumar P, Lakshmikuttyamma A (2016) Quercetin regulates β-catenin signaling and reduces the migration of triple negative breast cancer. Mol Carcinog 55:743–756. 10.1002/mc.22318 25968914 10.1002/mc.22318

[CR70] Timmer T, de Vries EG, de Jong S (2002) Fas receptor-mediated apoptosis: A clinical application? J Pathol 196:125–134. 10.1002/path.1028 11793363 10.1002/path.1028

[CR71] Tourneur L, Chiocchia G (2010) FADD: A regulator of life and death. Trends Immunol 31:260–269. 10.1016/j.it.2010.05.005 20576468 10.1016/j.it.2010.05.005

[CR72] Vargas AJ, Burd R (2010) Hormesis and synergy: Pathways and mechanisms of quercetin in cancer prevention and management. Nutr Rev 68:418–428. 10.1111/j.1753-4887.2010.00301.x 20591109 10.1111/j.1753-4887.2010.00301.x

[CR73] Walter P, Ron D (2011) The unfolded protein response: From stress pathway to homeostatic regulation. Science 334:1081–1086. 10.1126/science.1209038 22116877 10.1126/science.1209038

[CR74] Wang N, Zhao F, Lin P, Zhang G, Tang K, Wang A, Jin Y (2017) Knockdown of XBP1 by RNAi in mouse granulosa cells promotes apoptosis, inhibits cell cycle, and decreases estradiol synthesis. Int J Mol Sci 18:1152. 10.3390/ijms18061152 28555054 10.3390/ijms18061152 PMC5485976

[CR75] Wu S, Du R, Gao C, Kang J, Wen J, Sun T (2018) The role of XBP1s in the metastasis and prognosis of hepatocellular carcinoma. Biochem Biophys Res Commun 500:530–537. 10.1016/j.bbrc.2018.04.033 29627568 10.1016/j.bbrc.2018.04.033

[CR76] Yang D, Wang T, Long M, Li P (2020) Quercetin: Its main pharmacological activity and potential application in clinical medicine. Oxid Med Cell Longev 2020:8825387. 10.1155/2020/8825387 33488935 10.1155/2020/8825387 PMC7790550

[CR77] Zang C, Liu H, Bertz J, Possinger K, Koeffler HP et al (2009) Induction of endoplasmic reticulum stress response by TZD18, a novel dual ligand for peroxisome proliferator-activated receptor alpha/gamma, in human breast cancer cells. Mol Cancer Ther 8(8):2296–2307. 10.1158/1535-7163.MCT-09-0347 19671747 10.1158/1535-7163.MCT-09-0347

[CR79] Zhang X (2023) Molecular classification of breast cancer: Relevance and challenges. Arch Pathol Lab Med 147(1):46–51. 10.5858/arpa.2022-0070-RA 36136295 10.5858/arpa.2022-0070-RA

[CR78] Zhang L, Mu C, Zhang T, Wang Y, Wang Y et al (2020) Systemic delivery of aptamer-conjugated XBP1 siRNA nanoparticles for efficient suppression of HER2 + breast cancer. ACS Appl Mater Interfaces 12:32360–32371. 10.1021/acsami.0c07353 32613835 10.1021/acsami.0c07353

[CR80] Zhao Y, Zhang W, Huo M, Wang P, Liu X et al (2021) XBP1 regulates the protumoral function of tumor-associated macrophages in human colorectal cancer. Signal Transduct Target Ther 6:357. 10.1038/s41392-021-00761-7 34667145 10.1038/s41392-021-00761-7 PMC8526672

[CR81] Zhong Y, Li J, Wang JJ, Chen C, Tran JT et al (2012) X-box binding protein 1 is essential for the anti-oxidant defense and cell survival in the retinal pigment epithelium. PLoS ONE 7:e38616. 10.1371/journal.pone.0038616 22715395 10.1371/journal.pone.0038616 PMC3371004

[CR82] Zhou Y, Lee J, Reno CM, Sun C, Park SW et al (2011) Regulation of glucose homeostasis through a XBP-1-FoxO1 interaction. Nat Med 17(3):356–365. 10.1038/nm.2293 21317886 10.1038/nm.2293 PMC3897616

[CR83] Zhu J, Zhang X, Ai L, Yuan R, Ye J (2019) Clinicohistopathological implications of MMP/VEGF expression in retinoblastoma: A combined meta-analysis and bioinformatics analysis. J Transl Med 17:226. 10.1186/s12967-019-1975-3 31311559 10.1186/s12967-019-1975-3 PMC6636009

